# Histone H3 Lysine 18 Lactylation Promotes Cardiac Hypertrophy Through Activating GATA Binding Protein 4 Signaling

**DOI:** 10.1002/mco2.70421

**Published:** 2025-10-22

**Authors:** Mingzhu Wang, Zixian Liu, Yongbo Ma, Da Li, Yuan Lin, Yani Wang, Deyu Fu, Haidong Guo, Liang Hu

**Affiliations:** ^1^ Yueyang Hospital of Integrated Traditional Chinese and Western Medicine Shanghai University of Traditional Chinese Medicine Shanghai China; ^2^ Academy of Integrative Medicine Shanghai University of Traditional Chinese Medicine Shanghai China

**Keywords:** cardiac hypertrophy, histone lactylation, hypertension, transcription factor

## Abstract

Histone lactylation, particularly histone H3 lysine 18 lactylation (H3K18la), modulates gene expression profile in diverse cellular processes, which has emerged as a critical factor in cardiovascular disease pathogenesis. However, its specific role in cardiac hypertrophy remains unclear. This study investigates the mechanism of H3K18la in promoting cardiac hypertrophy using transverse aortic constriction‐induced mice model and a phenylephrine‐induced hypertrophic cardiomyocyte model. We found that elevated levels of Pan‐Kla and H3K18la were detected in hypertrophic left ventricular tissues and cardiomyocytes, accompanied by increased heart and left ventricle weights, enlarged cardiomyocyte cross‐sectional areas and heightened expression of ANP, BNP, and β‐MHC. Clinical observations revealed a positive correlation between serum lactate levels and hypertrophic cardiomyopathy in patients. Furthermore, inhibition of lactylation reversed these effects, suggesting a direct role of H3K18la in hypertrophic gene expression. Mechanistically, H3K18la was found to interact with GATA4, enhancing its transcriptional activity as demonstrated by increased ANP promoter activity. Moreover, suppression of GATA4 mitigated the hypertrophic response, highlighting its crucial role downstream of H3K18la. Our findings identify H3K18la lactylation as a novel epigenetic mechanism driving cardiac hypertrophy through GATA4 activation. This implicates potential therapeutic targets for hypertrophic heart diseases.

## Graphical abstract

Proposed model of the interaction of the H3K18la lactylation with GATA4 to promote the cardiac hypertrophy. The image was created using Home for Researchers.com.

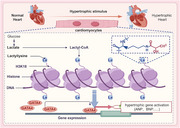

## 1 Introduction

Cardiac hypertrophy, characterized by an enlargement of the heart due to increased size and mass, represents a complex pathological response to chronic stressors such as hypertension, valve diseases, or myocardial infarction [1, 2]. Initially perceived as a compensatory mechanism to maintain cardiac function, prolonged hypertrophy often progresses to heart failure, a condition associated with significant morbidity and mortality worldwide [3, 4, 5, 6, 7, 8, 9]. Understanding the molecular mechanisms underlying hypertrophic growth is crucial for developing effective therapeutic strategies to manage cardiovascular diseases.

Emerging evidence now extends the functional relevance of protein lactylation to cardiovascular pathophysiology [10]. Recent studies have implicated lactylation in coronary heart disease, where it modulates metabolic reprogramming and oxidative stress responses in ischemic cardiomyocytes [11, 12]. Myocardial infarction models reveal that lactylation drives proinflammatory macrophage polarization and fibroblast activation, exacerbating postinfarction remodeling through nuclear factor kappa B (NF‐κB) and tansforming growth factor β (TGF‐β)pathways [13, 14, 15]. These findings position lactylation as a dynamic regulator of both adaptive and maladaptive processes in cardiovascular tissues [16, 17]. Moreover, recent advances in epigenetics have underscored the role of histone modifications as crucial regulators of gene expression patterns in both health and disease [18, 19, 20]. Histone lactylation, a relatively novel epigenetic modification involving the addition of lactate groups to lysine residues of histone proteins, has emerged as a critical player in diverse cellular processes [21]. This modification influences chromatin structure and gene accessibility, thereby impacting transcriptional outcomes in various cellular contexts [22]. Among the specific histone lactylation marks, H3K18la (lactylation of histone H3 at lysine 18) has garnered significant attention due to its potential role in modulating gene expression profiles in different cellular states [23, 24]. Previous studies have linked H3K18la to altered transcriptional programs in cancer cells and immune responses, suggesting its broader implications in regulating cellular functions beyond chromatin dynamics [25, 26, 27, 28, 29].

H3K18la is an epigenetic marker linked to diverse biological processes and disease states. Recent studies [30, 31] have discovered that in vascular endothelial cells, abnormally elevated aerobic glycolysis results in increased histone H3K18 lactylation, which in turn promotes the development of atherosclerosis. Meanwhile, enhanced glycolysis and increased lactate in vascular smooth muscle cells promote histone H3K18 lactylation, leading to the occurrence of arterial calcification. However, the specific mechanism of H3K18la in cardiomyocytes still requires further investigation. In the realm of cardiovascular biology, H3K18la has recently been implicated in cardiac diseases. This modification is hypothesized to contribute to the aberrant gene expression patterns associated with hypertrophic growth, potentially exacerbating pathological remodeling of the heart [32]. Specifically, the precise mechanisms by which H3K18 lactylation influences cardiac hypertrophy are not yet fully understood.

The transcription factor GATA Binding Protein 4 (GATA4) plays a pivotal role in cardiac development and function by orchestrating the expression of genes critical for cardiomyocyte growth, survival, and contractility [33]. GATA4 is known to interact with various epigenetic modifiers to modulate chromatin accessibility and transcriptional activity in response to physiological and pathological cues [34, 35, 36, 37]. Notably, dysregulation of GATA4 has been implicated in the progression of cardiac hypertrophy and heart failure, underscoring its potential as a therapeutic target in cardiovascular medicine [38]. Although the association between GATA4 and cardiovascular disease has been extensively studied, there is currently insufficient research on whether it regulates epigenetic modifications in myocardial cells, especially the role of H3K18la in myocardial hypertrophy.

Given the multifunctional nature of GATA4 as a transcription factor and the role of H3K18la in gene expression regulation, this study aims to elucidate the intricate interplay between H3K18la lactylation and GATA4 signaling pathways in the context of cardiac hypertrophy. Utilizing in vivo transverse aortic constriction (TAC) and in vitro cardiomyocyte cultures, we demonstrated that H3K18la enhances GATA4 transcriptional activity, thereby promoting the expression of hypertrophic markers and exacerbating pathological cardiac remodeling. By integrating biochemical, molecular biology, and physiological approaches, this research aims to uncover novel insights into how epigenetic modifications, particularly H3K18la, influence GATA4‐dependent transcriptional regulation in hypertrophic cardiomyocytes. The outcomes of this study are poised to deepen our understanding of the pathogenesis of cardiac hypertrophy and may open avenues for therapeutic interventions targeting epigenetic mechanisms in cardiovascular diseases.

Subsequent sections will detail the experimental methodologies employed, the results obtained, and the implications of our findings for both fundamental science and clinical cardiology. This comprehensive investigation seeks to bridge fundamental epigenetic research with translational applications in cardiovascular medicine, with the goal of advancing therapeutic strategies for cardiac hypertrophy and related conditions.

## 2 Results

### 2.1 The Levels of Pan‐Kla and H3K18la were Elevated in the Left Ventricle Tissues from TAC‐Induced Hypertrophy Mice

**FIGURE 1:**
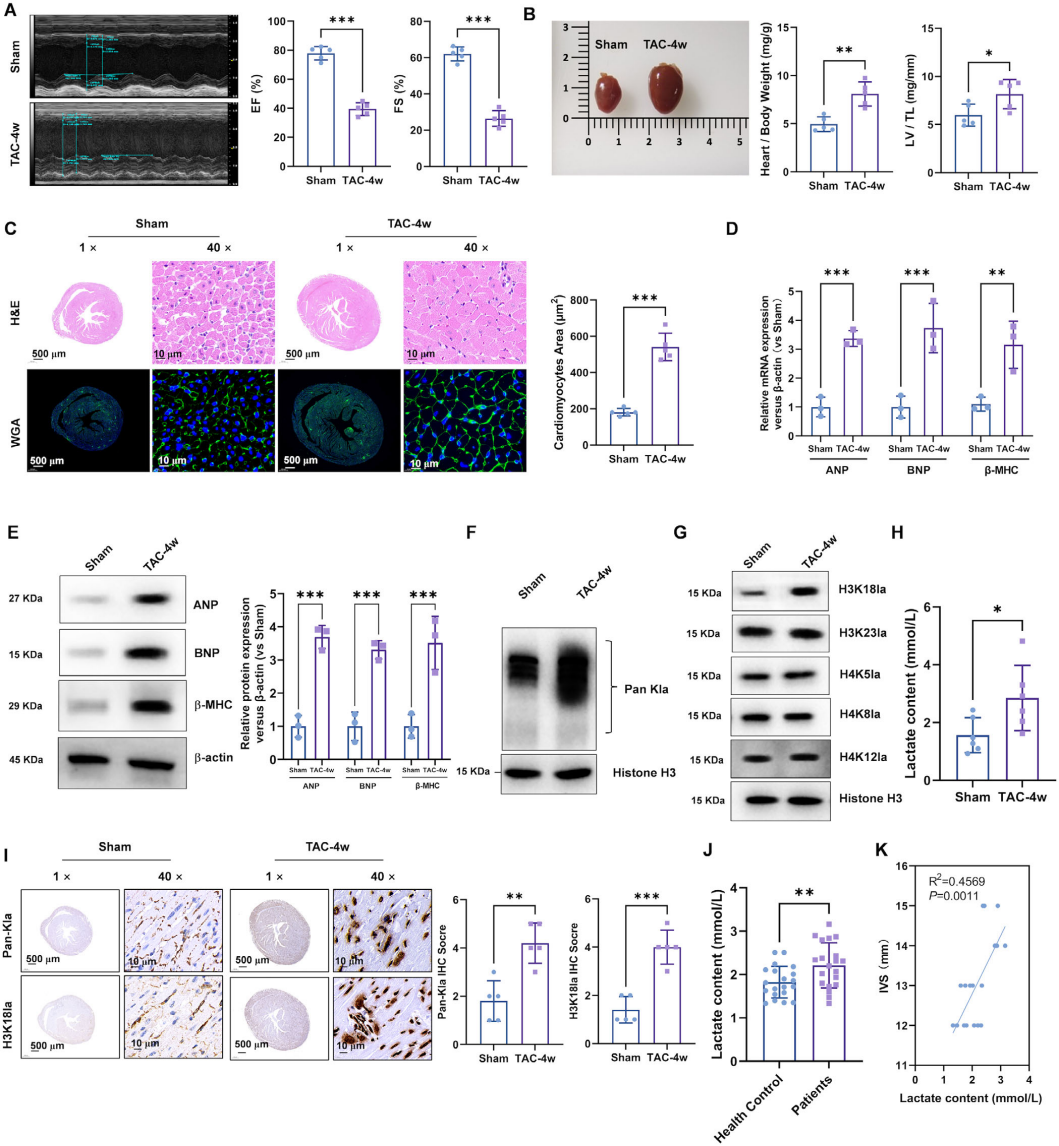
The levels of Pan‐Kla and H3K18la were elevated in the left ventricle tissues from TAC‐induced hypertrophy mice. (A) Representative examples of M‐mode echocardiograms of hearts from TAC or a sham operation. (B) The ratio of heart weight to body weight (HW/BW). The ratio of left ventricle weight to tibia length (LV/TL). (C) H&E and WGA staining of heart sections from TAC and sham mice was used to quantify the cross‐sectional areas of the cardiomyocytes (1× scale bar: 500 µm; 40× scale bar: 10 µm). (D) Real‐time PCR and (E) Western blotting was performed to analysis of ANP, BNP, and β‐MHC mRNA and protein expression, respectively. Relative to sham group. (F) Western blotting was performed to analysis of Pan‐Kla protein expression. (G) Western blotting was performed to analysis of H3K18la, H3K23la, H4K5la, H4K8la, and H4K12la protein expression. (H) The levels of serum lactate in the serum of TAC‐induced hypertrophy mice sham mice were detected by colorimetric assay kit. (I) The immunohistochemistry staining was performed to analysis of Pan‐Kla and H3K18la expression. (J) The levels of serum lactate in the serum of hypertrophy patients and health controls were detected by colorimetric assay kit. (K) High levels of lactate were positive corelative with high interventricular septal thickness.

To investigate the role of H3K18la lactylation in cardiac hypertrophy, we employed TAC‐induced hypertrophy, which mimic chronic pressure overload and adrenergic stimulation during cardiac remodeling. Echocardiographic analysis revealed that TAC mice showed a significant cardiac remodeling characterized by increased left ventricular (LV) mass and reduced cardiac function compared with sham‐operated mice (Figure 1A). The increased LV end‐diastolic volume, LV end‐systolic volume, LV internal dimension‐diastole, LV internal dimension‐systole, cardiac output, and decreased stroke volume were also shown in the TAC mice (Figure S1). Consistent with hypertrophic growth, TAC mice also exhibited elevated ratios of heart weight to body weight (HW/BW) and LV weight to tibia length (LV/TL) (Figure 1B). Histological examination of heart sections stained with hematoxylin and eosin (H&E) and wheat germ agglutinin (WGA) further revealed enlarged cardiomyocytes and increased cross‐sectional areas in TAC mice (Figure 1C). qPCR and Western blotting analyses demonstrated the upregulated expression of hypertrophic markers including atrial natriuretic peptide (ANP), brain natriuretic peptide (BNP), and β myosin heavy chain (β‐MHC) at both mRNA (Figure 1D) and protein levels (Figure 1E).

Importantly, we observed increased protein expression of pan‐lysine lactylation (Pan‐Kla) and specifically H3K18la in TAC hearts compared with sham mice (Figure 1F, G). Consistently, the levels of lactate in the serum of TAC mice were significantly higher compared with sham mice (Figure 1H). Immunohistochemistry staining further confirmed elevated levels of Pan‐Kla and H3K18la in hypertrophic hearts, which may regulate gene expression contributing pathological cardiac remodeling (Figure 1I).

To investigate H3K18la lactylation in cardiac hypertrophy, we also evaluated the lactate levels in serum from hypertrophic cardiomyopathy patients and health donors. As shown in Figure 1J, the serum lactate levels in hypertrophic cardiomyopathy patients were higher compared with healthy donors. Moreover, there is a positive correlation between elevated lactate levels and increased interventricular septal thickness in Figure 1K, suggesting a potential link between lactate and cardiac hypertrophy progression.

### 2.2 The Levels of Pan‐Kla and H3K18la were Elevated in the Phenylephrine‐Induced Hypertrophy

**FIGURE 2:**
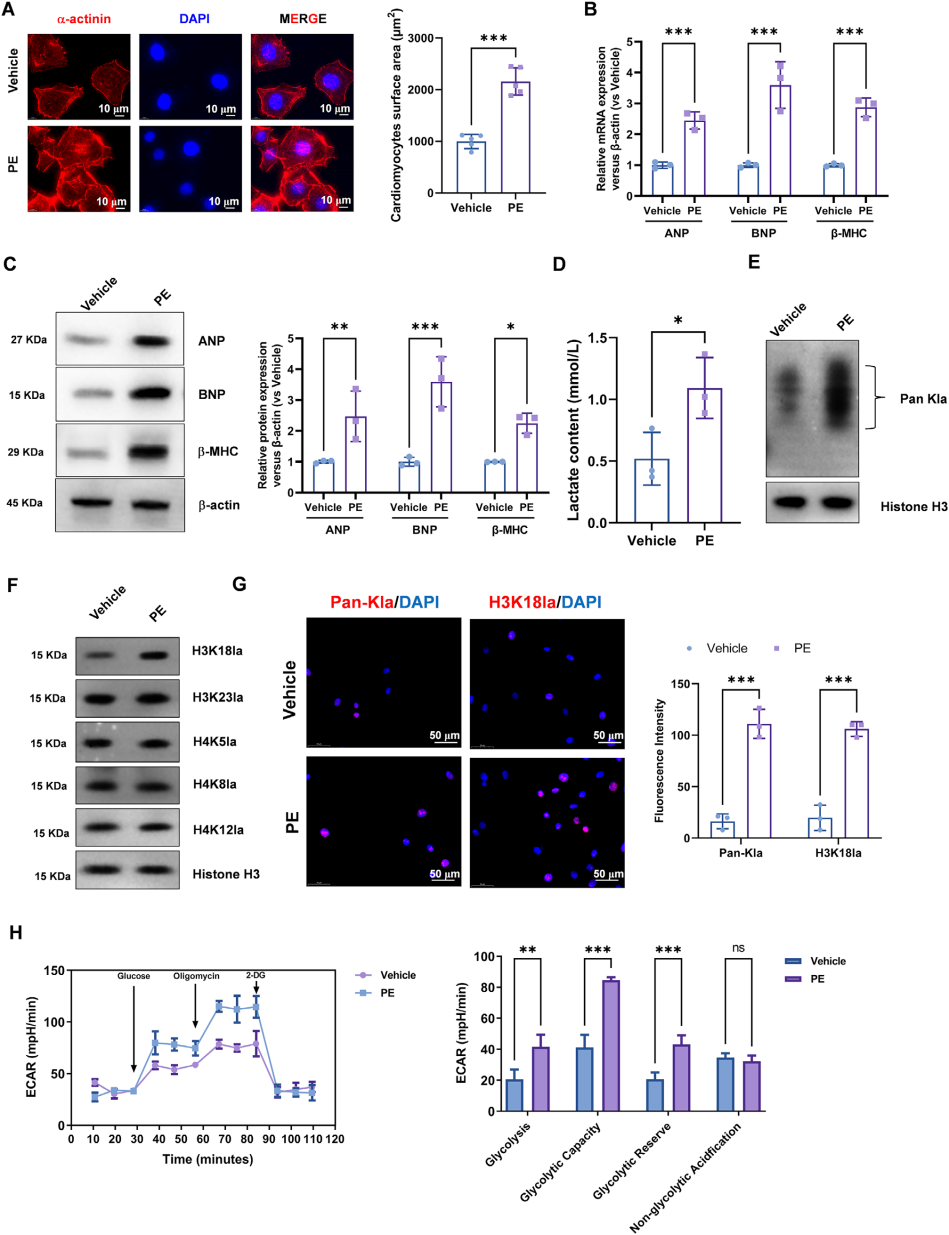
The levels of Pan‐Kla and H3K18la were elevated in the PE‐induced hypertrophy. (A) Representative photograph of cardiomyocytes treated with 100 mM PE. Sarcomeric organization was visualized by α‐actinin staining. Scale bar, 10 mM. Cell‐surface areas were measured by using NIH ImageJ software (*n* = 100 cells per group). (B) Real‐time PCR and (C) Western blotting was performed to analysis of ANP, BNP, and β‐MHC mRNA and protein expression, respectively. Relative to vehicle group. (D) The levels of serum lactate in the culture medium of PE‐induced hypertrophic cardiomyocytes and vehicle cardiomyocytes were detected by colorimetric assay kit. (E) Western blotting was performed to analysis of Pan‐Kla protein expression. (F) Western blotting was performed to analysis of H3K18la, H3K23la, H4K5la, H4K8la, and H4K12la protein expression. (G) The immunofluorescence staining was performed to analysis of Pan‐Kla and H3K18la expression. (H) Seahorse analyzer was used to analysis the glycolytic metabolism in vehicle and PE‐stimulated cardiomyocytes.

Next, we utilized phenylephrine (PE) treatment to induce hypertrophy in cultured cardiomyocytes. Treatment with 100 mM PE resulted in significant cellular enlargement, as evidenced by increased cell surface area measured using NIH ImageJ software (Figure 2A). Consistent with the TAC model, PE‐stimulated cardiomyocytes exhibited the increased expression of ANP, BNP, and β‐MHC at both mRNA (Figure 2B) and protein levels (Figure 2C). The levels of lactate in the culture medium of PE‐induced hypertrophic cardiomyocytes were dramatically higher compared with vehicle cardiomyocytes (Figure 2D). Consistent with the in vivo results, we observed the increased protein expression of Pan‐Kla and specifically H3K18la, while there is no obvious expression change of histone H3 lysine 23 lactylation (H3K23la), histone H4 lysine 5 lactylation (H4K5la), histone H4 lysine 8 lactylation (H4K8la), and histone H4 lysine 12 lactylation (H4K12la) in PE‐stimulated cardiomyocytes compared with vehicle cardiomyocytes (Figure 2E, F).

Immunofluorescence staining showed the Pan‐Kla and H3K18la were increased in PE‐treated cardiomyocytes compared with untreated cardiomyocytes, suggesting a conserved role for H3K18la in hypertrophic gene regulation across different models (Figure 2G). Furthermore, PE‐stimulated cardiomyocytes exhibited elevated glycolytic metabolism using the Seahorse analyzer (Figure 2H).

### 2.3 Inhibition of Lactylation Attenuated the TAC Impaired Cardiac Contractile Functions and Induced Hypertrophy

**FIGURE 3:**
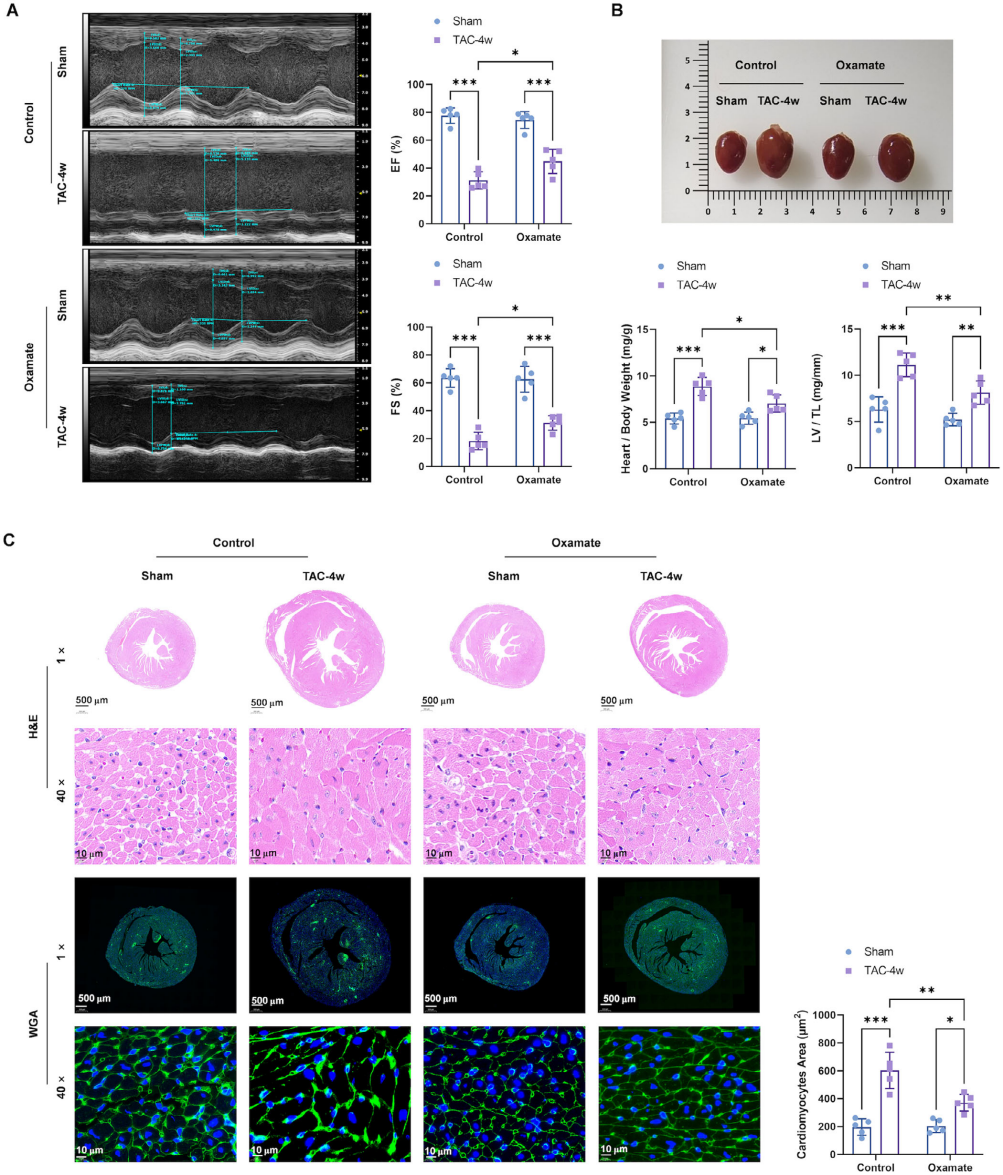
Inhibition of lactylation attenuated the TAC impaired cardiac contractile functions and induced hypertrophy mice. (A) Representative examples of M‐mode echocardiograms of hearts from TAC and oxamate treatment. (B) The ratio of heart weight to body weight (HW/BW. The ratio of left ventricle weight to tibia length (LV/TL). (C) H&E and WGA staining of heart sections from TAC and sham mice was used to quantify the cross‐sectional areas of the cardiomyocytes (1× scale bar: 500 µm; 40× scale bar: 10 µm).

**FIGURE 4:**
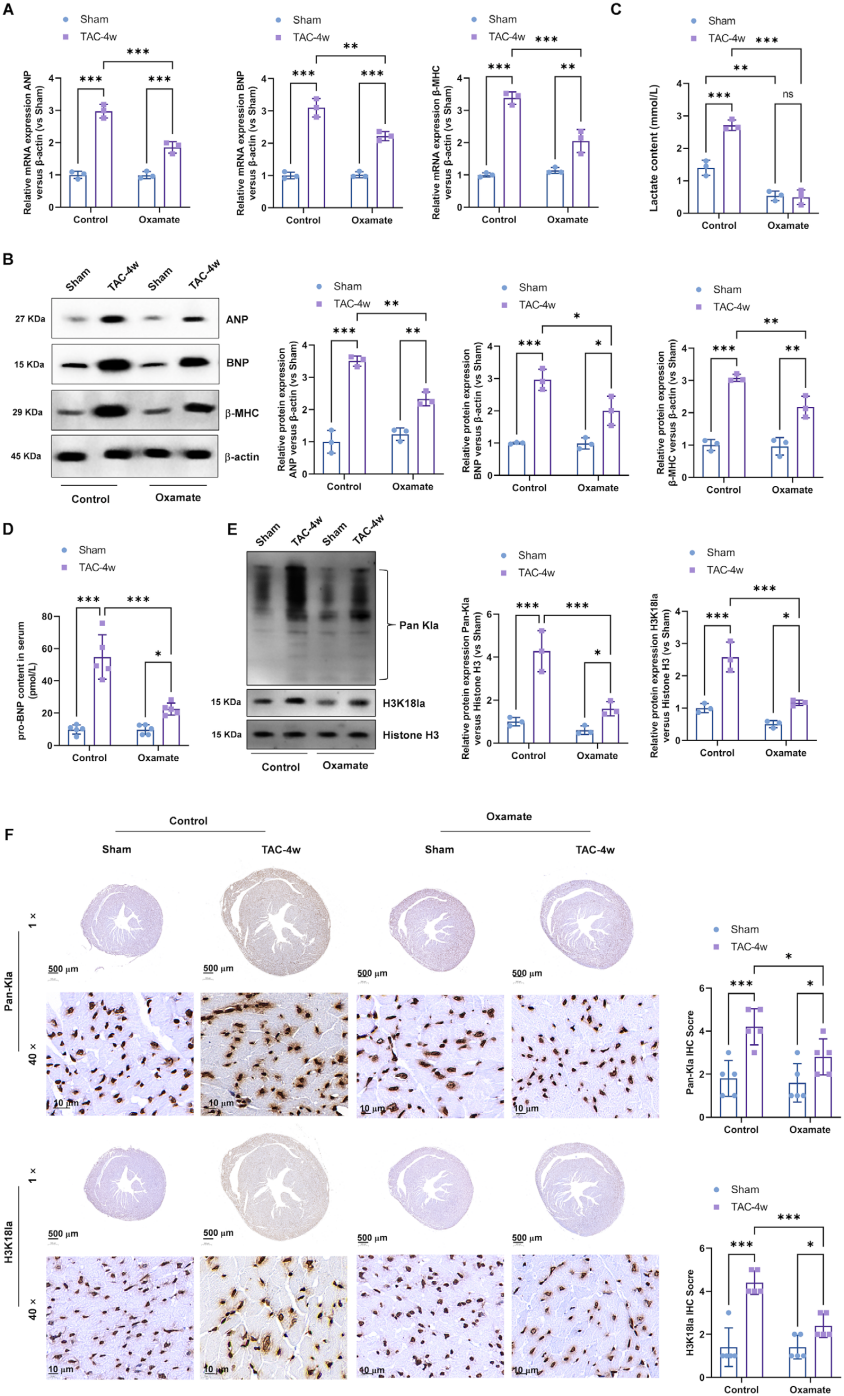
Inhibition of lactylation inhibits the expression of TAC elevated hypertrophic genes. (A) Real‐time PCR and (B) Western blotting was performed to analysis of ANP, BNP, and β‐MHC mRNA and protein expression, respectively. Relative to sham group. (C) The levels of serum lactate and (D) pro‐BNP in the serum of TAC‐induced hypertrophy mice and sham mice were detected by colorimetric assay kit. (E) Western blotting was performed to analysis of Pan‐Kla protein and H3K18la expression. Relative to sham group. (F) The immunohistochemistry staining was performed to analysis of Pan‐Kla and H3K18la expression.

To elucidate the function of H3K18la lactylation in cardiac hypertrophy, we pharmacologically inhibited lactylation using specific inhibitors, oxamate, which is a lactylation inhibitor, through inhibiting the activity of lactate dehydrogenase A. We also evaluated their effects on hypertrophic markers and cardiac function in both TAC and PE‐induced models. Lactylation inhibitors attenuated TAC‐induced cardiac hypertrophy as evidenced by improved cardiac function assessed via echocardiography (Figures 3A and S2), reduced HW/BW and LV/TL ratios (Figure 3B), and normalized cardiomyocyte cross‐sectional areas (Figure 3C). Molecular analysis revealed that lactylation inhibition significantly decreased the expression of ANP, BNP, and β‐MHC at both mRNA (Figure 4A) and protein levels (Figure 4B). Moreover, lactylation inhibition significantly decreased the levels of lactate (Figure 4C) and pro‐BNP (Figure 4D) in the serum of TAC mice. Western blotting (Figure 4E) and Immunohistochemistry staining (Figure 4F) demonstrated levels of Pan‐Kla and H3K18la reduced after lactylation inhibitors treatment, which confirmed the lactylation inhibition efficacy of oxamate in vivo.

### 2.4 Inhibition of Lactylation Inhibits the PE‐Induced Hypertrophy

**FIGURE 5:**
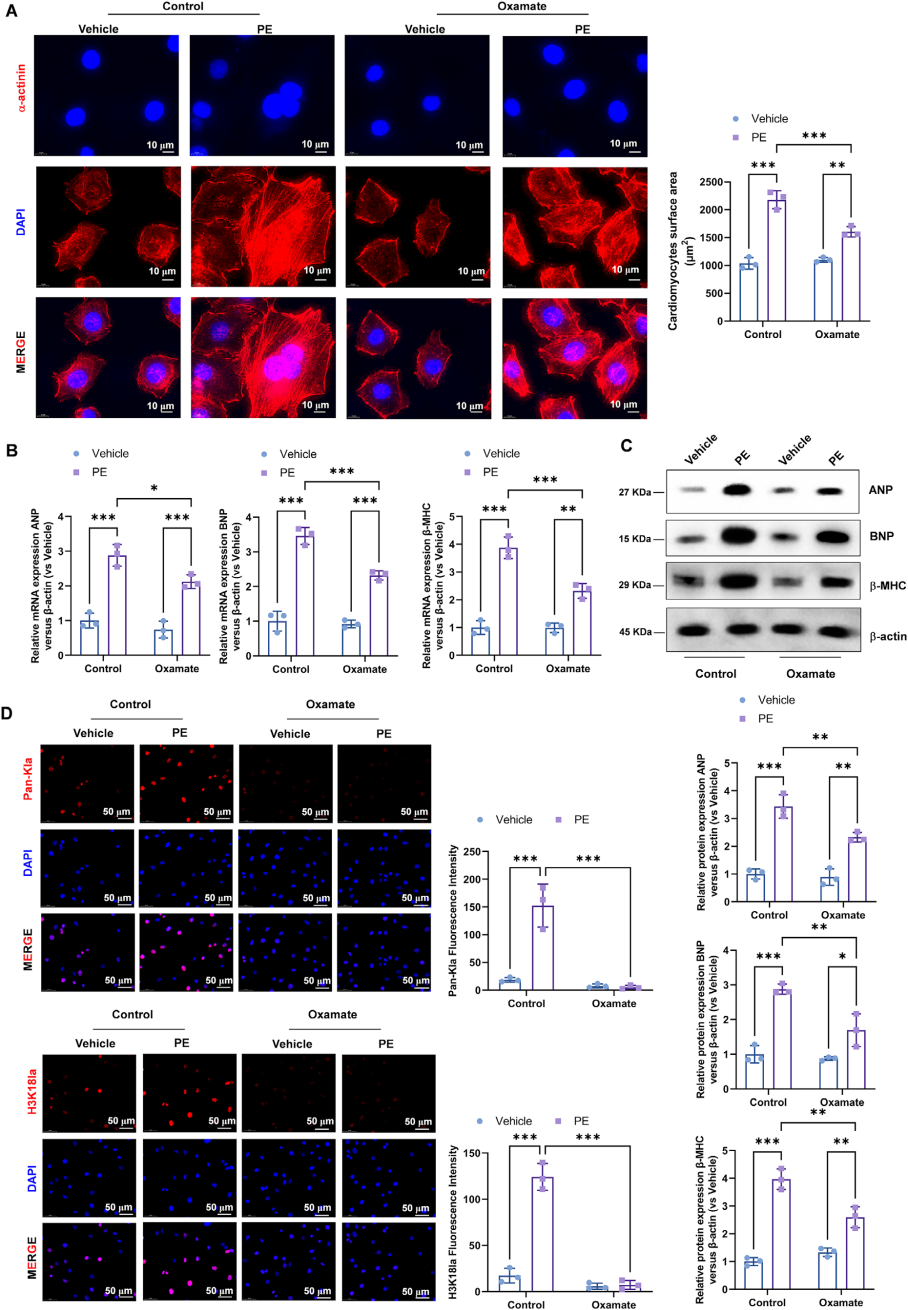
Inhibition of lactylation inhibits the PE‐induced hypertrophy. (A) Representative photograph of cardiomyocytes treated with 100 mM PE. Sarcomeric organization was visualized by α‐actinin staining. Scale bar, 10 µm. Cell‐surface areas were measured by using NIH ImageJ software (*n* = 100 cells per group). (B) Real‐time PCR and (C) Western blotting was performed to analysis of ANP, BNP, and β‐MHC mRNA and protein expression, respectively. Relative to vehicle group. (D) The immunofluorescence staining was performed to analysis of Pan‐Kla and H3K18la expression.

Similarly, lactylation inhibition attenuated PE‐induced hypertrophy in cultured cardiomyocytes. Treatment with lactylation inhibitors reduced cell surface area (Figure 5A) and suppressed the expression of hypertrophic markers ANP, BNP, and β‐MHC at both mRNA (Figure 5B) and protein levels (Figure 5C). Immunofluorescence staining revealed decreased levels of Pan‐Kla and H3K18la in inhibitor‐treated cardiomyocytes compared with PE‐stimulated controls, indicating a direct role of H3K18la in regulating hypertrophic gene expression in vitro (Figure 5D).

### 2.5 H3K18 Lactylation Interacted with the GATA4 Protein and Enhanced its Transcription Activity

**FIGURE 6:**
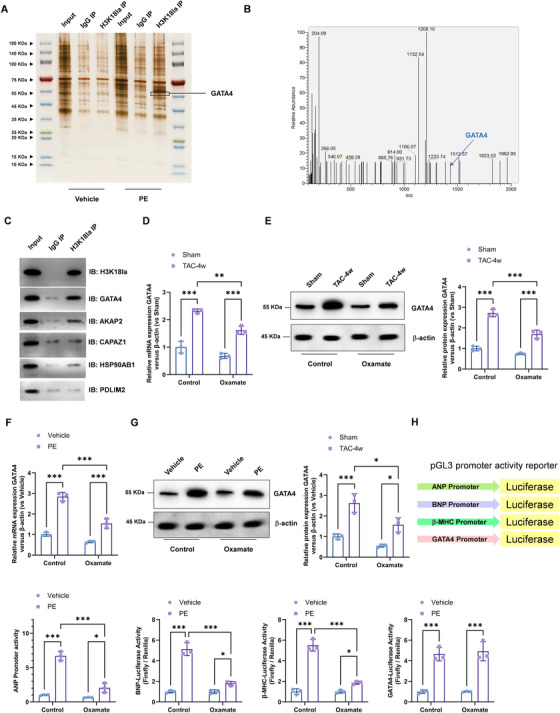
H3K18 lactylation interacted with the GATA4 protein and enhanced its transcription activity. (A) Silver staining of the SDS‐PAGE of H3K18la Co‐IP proteins and (B) LC/MS identified the potential proteins that interacts with H3K18la. (C) Western blotting verified the interaction between H3K18la and potential targets (GATA4, AKAP2, CAPAZ1, HSP90AB1, and PDLIM2). (D) Real‐time PCR and (E) Western blotting was performed to analysis of GATA4 mRNA and protein expression in heart tissues, respectively. Relative to sham group. (F) Real‐time PCR and (G) Western blotting was performed to analysis of GATA4 mRNA and protein expression in cardiomyocytes, respectively. Relative to vehicle group. (H) Dual‐luciferase assay was performed to analysis the promoter activity of ANP, BNP, β‐MHC, and GATA4.

To explore the molecular mechanism underlying H3K18la‐mediated hypertrophic gene expression, we investigated the interaction between H3K18la and GATA4, a key transcription factor involved in cardiac hypertrophy. Co‐immunoprecipitation (Co‐IP) followed by mass spectrometry identified GATA4 as a potential interacting partner of H3K18la in cardiac tissues (Figure 6A, B). Western blotting verified the interaction between H3K18la and potential targets, including GATA4, A kinase (PRKA) anchor protein 2 (AKAP2), α1 capping protein (actin filament) muscle Z‐line, alpha 1(CAPAZ1), heat shock protein 90 kDa alpha class B member 1 (HSP90AB1), and PDZ and LIM domain protein 2 (PDLIM2), which showed GATA4 and AKAP2 were highly enriched in the H3K18la IP group (Figure 6C). Real‐time PCR and Western blotting confirmed the increased levels of GATA4 in both heart tissues from TAC mice (Figure 6D, E) and cultured cardiomyocytes from PE‐stimulated cardiomyocytes (Figure 6F, G) was suppressed by oxamate treatment. Moreover, PE treatment significantly enhanced ANP, BNP, β‐MHC, and GATA4 transcriptional activity as assessed by dual‐luciferase assay using ANP, BNP, β‐MHC, and GATA4 promoter constructs (Figure 6H), suggesting that H3K18la lactylation promotes hypertrophic gene expression via activation of GATA4‐dependent transcriptional programs.

### 2.6 Inhibition of Lactylation Attenuated the TAC Impaired Cardiac Contractile Functions and Induced Hypertrophy Mice via the Suppression of GATA4

**FIGURE 7:**
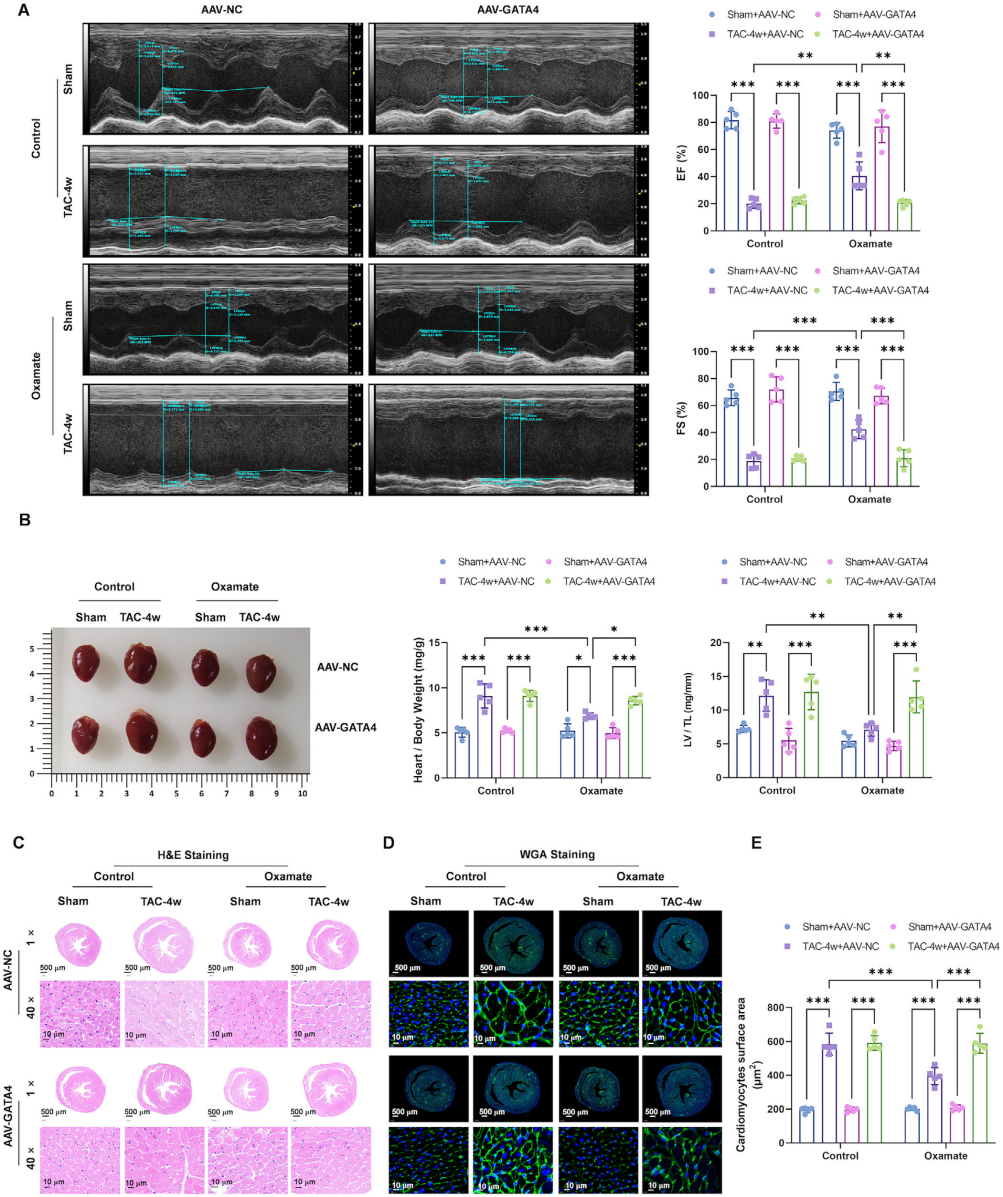
Inhibition of lactylation attenuated the TAC impaired cardiac contractile functions and induced hypertrophy mice via the suppression of GATA4. (A) Representative examples of M‐mode echocardiograms of hearts from TAC, oxamate, and AAV‐GATA4 treatment. (B) The ratio of heart weight to body weight (HW/BW). The ratio of left ventricle weight to tibia length (LV/TL). (C–E) H&E (C) and WGA (D) staining of heart sections from TAC and sham mice was used to quantify the cross‐sectional areas of the cardiomyocytes (E). 1× Scale bar: 500 µm; 40× scale bar: 10 µm.

To further validate the role of AKAP2 and GATA4 in lactylation‐mediated hypertrophy, we overexpressed AKAP2 and GATA4 using adeno‐associated virus (AAV) and assessed its effects on hypertrophic markers in both TAC and PE‐induced models. Overexpression of AKAP2 did not affect the lactylation inhibition attenuated cardiac hypertrophy in TAC mice (Figure S3). However, overexpression of GATA4 expression significantly reversed the lactylation inhibition attenuated cardiac hypertrophy in TAC mice, as evidenced by decreased cardiac function (Figures 7A and S4), increased HW/BW and LV/TL ratios (Figure 7B), and normalized cardiomyocyte sizes by H&E (Figure 7C) and WGA (Figure 7D, E) staining of heart sections.

### 2.7 Inhibition of Lactylation Inhibits the PE‐Induced Hypertrophy via the Suppression of GATA4

**FIGURE 8:**
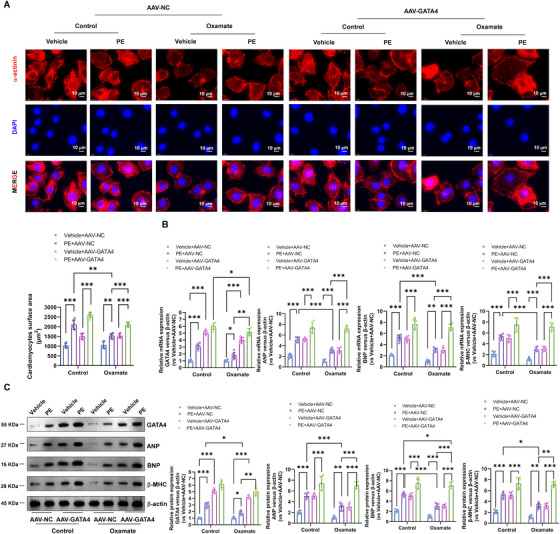
Inhibition of lactylation inhibits the PE‐induced hypertrophy via the suppression of GATA4. (A) Representative photograph of cardiomyocytes treated with 100 mM PE. Sarcomeric organization was visualized by α‐actinin staining. Scale bar, 10 µm. Cell‐surface areas were measured by using NIH ImageJ software (*n* = 100 cells per group). (B) Real‐time PCR and (C) Western blotting was performed to analysis of GATA4, ANP, BNP, and β‐MHC mRNA and protein expression, respectively. Relative to vehicle + AAV‐NC group.

Similarly, inhibition of lactylation with oxamate attenuated PE‐induced hypertrophy in cultured cardiomyocytes. Treatment with oxamate reduced cell surface area (Figure 8A) and suppressed the expression of hypertrophic markers ANP, BNP, and β‐MHC at both mRNA (Figure 8B) and protein levels (Figure 8C). However, overexpression of GATA4 expression significantly reversed the lactylation inhibition attenuated PE‐induced hypertrophy in cultured cardiomyocytes, including the increased cell surface area (Figure 8A) and induced expression of hypertrophic markers ANP, BNP, and β‐MHC at both mRNA (Figure 8B) and protein levels (Figure 8C).

Overall, our results demonstrate that H3K18la lactylation promotes cardiac hypertrophy through activation of GATA4 signaling pathways. Mechanistically, H3K18la interacts with GATA4 to enhance its transcriptional activity, thereby promoting the expression of hypertrophic markers and exacerbating pathological cardiac remodeling in response to pressure overload and adrenergic stimulation. These findings provide novel insights into the epigenetic regulation of cardiac hypertrophy and highlight potential therapeutic targets for mitigating hypertrophic growth and improving cardiac function in patients with cardiovascular diseases.

## 3 Discussion

The present study aimed to elucidate the role of H3K18la lactylation in cardiac hypertrophy through GATA4 signaling pathways using both in vivo and in vitro models. Our results provide compelling evidence that H3K18la lactylation plays a critical role in promoting hypertrophic gene expression and exacerbating pathological cardiac remodeling in response to pressure overload and adrenergic stimulation.

Epigenetic modifications, including histone lactylation, have emerged as pivotal regulators of gene expression in various biological processes, including cardiovascular diseases [39, 40, 41, 42]. Our findings contribute to the growing body of evidence suggesting that histone modifications can dynamically influence cardiac gene expression patterns under stress conditions. Histone lactylation, specifically H3K18la, was significantly elevated in both the TAC and PE‐induced models of cardiac hypertrophy. This modification correlated with increased expression of hypertrophic markers such as ANP, BNP, and β‐MHC at both mRNA and protein levels. These observations underscore the importance of epigenetic mechanisms in driving the pathological remodeling of the heart in response to different stressors.

A key mechanistic finding of our study is the interaction between H3K18la and GATA4, a crucial transcription factor known to regulate cardiac hypertrophy [43, 44, 45, 46]. The specificity of GATA4 in regulating hypertrophy‐related genes is supported by extensive evidence from prior studies. GATA4 has been well‐established as a direct transcriptional activator of hallmark cardiac hypertrophy markers, including ANP, BNP, and β‐MHC [36]. These genes encode proteins critical to pathological cardiac remodeling—ANP and BNP serve as molecular markers of hemodynamic stress, while β‐MHC contributes to sarcomeric contractility changes during hypertrophy [38]. Mechanistically, chromatin immunoprecipitation assays in earlier work demonstrated GATA4 binding to conserved promoter/enhancer regions of these genes, directly driving their transcription in response to prohypertrophic stimuli [35]. Importantly, genetic ablation or pharmacological inhibition of GATA4 attenuates the induction of these genes and suppresses hypertrophic growth in vivo and in vitro [33]. Our findings align with this paradigm, as H3K18la‐enhanced GATA4 activity selectively amplified ANP promoter‐driven transcription in our luciferase assays. In our study, Co‐IP experiments coupled with mass spectrometry identified GATA4 as a binding partner of H3K18la in cardiac tissues and cultured cardiomyocytes. This interaction was further validated by Western blotting, demonstrating physical association between H3K18la and GATA4. Moreover, functional assays revealed that H3K18la enhances GATA4 transcriptional activity, as evidenced by increased ANP promoter activity in luciferase reporter assays. These findings suggest a direct mechanistic link between H3K18la lactylation and GATA4‐dependent hypertrophic gene expression programs. This consistency across experimental models reinforces the premise that GATA4 operates as a nodal regulator of hypertrophy‐related gene programs, rather than exerting indirect or pleiotropic effects. The convergence of H3K18 lactylation with this established transcriptional axis provides a plausible epigenetic mechanism for fine‐tuning GATA4‐dependent pathological gene expression.

Understanding the molecular mechanisms underlying cardiac hypertrophy is crucial for developing targeted therapies to mitigate pathological remodeling and improve cardiac function in patients with cardiovascular diseases [47, 48, 49, 50]. Our study identifies H3K18la lactylation as a potential therapeutic target for intervention in hypertrophic cardiomyopathy. Pharmacological inhibition of histone lactylation using specific inhibitors attenuated hypertrophic responses in both in vivo and in vitro models. Treatment with lactylation inhibitors not only improved cardiac function but also reduced the expression of hypertrophic markers and normalized cardiomyocyte morphology. These results highlight the therapeutic potential of targeting histone lactylation pathways to counteract pathological cardiac remodeling. Future studies should focus on further elucidating the specific molecular mechanisms by which H3K18la regulates GATA4 activity and hypertrophic gene expression. Additionally, investigating the broader epigenetic landscape in cardiac hypertrophy, including other histone modifications and chromatin remodeling complexes, may provide deeper insights into the complex regulatory networks governing cardiac gene expression under stress conditions [51, 52, 53].

While our study provides compelling evidence for the role of H3K18la lactylation in cardiac hypertrophy, several limitations should be acknowledged. Although we utilized both murine models and cultured cardiomyocytes to study hypertrophic responses, extrapolating these findings to human cardiac pathology requires cautious interpretation. Constrained by the design of this study, we mainly assessed the effect on the lactylation inhibition attenuated cardiac hypertrophy in TAC mice and cardiomyocytes. In subsequent experiments, we intend to prolong the intervention period to further elucidate the role of GATA4 in myocardial hypertrophy [54]. Future translational studies using human cardiac tissues or patient‐derived induced pluripotent stem cell‐derived cardiomyocytes could validate our findings in a clinical context. Second, while our study focused on H3K18la, other lysine lactylation sites on histones (e.g., H4K5la, H4K8la, H4K12la) were also observed to be modulated under hypertrophic conditions. Investigating the interplay between these different histone lactylation marks and their combinatorial effects on cardiac gene expression warrants further investigation.

In conclusion, our study provides novel insights into the epigenetic regulation of cardiac hypertrophy through H3K18la lactylation‐mediated activation of GATA4 signaling pathways. By identifying histone lactylation as a critical regulator of hypertrophic gene expression, we highlight potential therapeutic strategies for mitigating pathological cardiac remodeling and improving outcomes for patients with cardiovascular diseases. Future efforts aimed at dissecting the intricate epigenetic mechanisms in cardiac pathology will undoubtedly pave the way for innovative therapeutic interventions in the field of cardiovascular medicine.

## 4 Materials and Methods

### 4.1 Serum Lactate Analysis

The serum from health donors and hypertrophic cardiomyopathy patients were collected from the patients (Yueyang Hospital of Integrated Traditional Chinese and Western Medicine, Shanghai University of Traditional Chinese Medicine. 2022–051). Written informed consent was obtained from all participants before inclusion in the study. We recruited 41 patients with hypertensive myocardial hypertrophy aged between 18 and 85 years. For the hypertensive myocardial hypertrophy group, the inclusion criteria included: (1) a blood pressure reading greater than 140/90 mmHg; (2) the Sokolow–Lyon voltage on an electrocardiogram is greater than 3.8 mV, or the LV mass index on echocardiography is ≥109 g/m^2^ for men and ≥105 g/m^2^ for women. The exclusion criteria included: (1) hypertrophic cardiomyopathy; (2) severe renal insufficiency; (3) liver injury; (4) acute infection; (5) neoplasm; (6) hematologic disorders; (7) immunosuppression (acquired immune deficiency syndrome, chemotherapy, high doses of immunosuppressive agents). Among the 41 hypertensive myocardial hypertrophy patients, 21 were excluded and 20 patients were included. The baseline characteristics of the analyzed patients are presented in Table S1.

Serum lactate levels were measured using a colorimetric assay kit according to the manufacturer's instructions. Briefly, serum samples from hypertrophic cardiomyopathy patients and healthy donors were collected, and lactate levels were determined by spectrophotometric analysis.

### 4.2 TAC Model

Adult male C57BL/6 mice (8–10 weeks old) were used for the TAC‐induced cardiac hypertrophy model. Animals were housed under standard conditions with a 12‐h light/dark cycle and free access to food and water. Surgical procedures were performed under sterile conditions. Briefly, mice were anesthetized with isoflurane (2–3% induction, 1–2% maintenance) and placed on a heating pad to maintain body temperature. A midline incision was made, and the transverse aorta was ligated between the innominate and left common carotid arteries using a 27‐gauge needle against a 27‐gauge blunt needle as described previously [8]. Sham‐operated mice underwent the same surgical procedures except for aortic constriction. After surgery, mice received analgesics (buprenorphine, 0.1 mg/kg) and were monitored until recovery. Echocardiography was performed using a high‐resolution imaging system (Vevo 770; VisualSonics) before and after TAC surgery to confirm the development of cardiac hypertrophy.

### 4.3 PE‐Induced Hypertrophy

Primary cultures of neonatal rat ventricular myocytes (NRVMs) were used to study the effects of adrenergic stimulation‐induced hypertrophy in vitro. NRVMs were isolated from 1 to 2‐day‐old Sprague–Dawley rat pups as previously described [9]. Cells were cultured in Dulbecco's modified Eagle's medium supplemented with 10% fetal bovine serum, penicillin (100 U/mL), and streptomycin (100 µg/mL). To induce hypertrophy, NRVMs were treated with 100 µM PE for 48 h.

### 4.4 Echocardiography

Cardiac function was assessed using transthoracic echocardiography (Vevo 770, VisualSonics) with a 30‐MHz probe. Mice were anesthetized with 1–2% isoflurane, and images were obtained in M‐mode and B‐mode from the parasternal short‐axis view at the level of the papillary muscles. LV dimensions and wall thickness were measured, and fractional shortening and ejection fraction were calculated to evaluate cardiac contractile function.

### 4.5 Histological Analysis

Heart tissues were fixed in 10% formalin, embedded in paraffin, and sectioned. Sections were stained with H&E for general morphology and WGA to measure cardiomyocyte cross‐sectional area. Images were captured using a light microscope.

### 4.6 RNA Extraction and Real‐time PCR

Total RNA was extracted from heart tissues or cultured cells using TRIzol reagent according to the manufacturer's instructions. RNA concentration and purity were determined spectrophotometrically (NanoDrop; Thermo Fisher Scientific). Reverse transcription was performed using a high‐capacity complementary DNA (cDNA) reverse transcription kit (Applied Biosystems) to synthesize cDNA. Real‐time PCR was carried out using SYBR Green Master Mix (Applied Biosystems) on a real‐time PCR system (Applied Biosystems 7500 Fast Real‐Time PCR System). Specific primers for hypertrophic markers (ANP, BNP, β‐MHC) and reference genes (GAPDH or β‐actin) were used. Relative mRNA expression levels were calculated using the ΔΔCt method. The primer used in this study were listed in Table S2.

### 4.7 Protein Extraction and Western Blotting

Protein extraction from heart tissues or cells was performed using RIPA buffer supplemented with protease and phosphatase inhibitors. Protein concentration was determined using the Bradford assay (Bio‐Rad). Equal amounts of protein were separated by SDS‐PAGE and transferred onto PVDF membranes. Membranes were blocked with 5% nonfat milk or BSA and incubated overnight at 4°C with primary antibodies against ANP (27426‐1‐AP; Proteintech, Wuhan, China), BNP (A2179; Abclonal, Shanghai, China), β‐MHC (A2179; Abclonal), GATA4 (19530‐1‐AP; Proteintech), and H3K18la (PA5‐116896; ThermoFisher, USA). After washing, membranes were incubated with HRP‐conjugated secondary antibodies. Protein bands were visualized using an enhanced chemiluminescence detection system (GE Healthcare) and quantified using densitometry analysis (Image J software).

### 4.8 Immunohistochemistry and Immunofluorescence Staining

Paraffin‐embedded heart sections or cultured cardiomyocytes were deparaffinized, rehydrated, and subjected to antigen retrieval. Sections were incubated with primary antibodies against Pan‐Kla, H3K18la, and GATA4, followed by appropriate secondary antibodies conjugated with either horseradish peroxidase (for immunohistochemistry) or fluorescent dyes (for immunofluorescence). Images were acquired using a fluorescence microscope.

### 4.9 Co‐IP

For Co‐IP experiments, heart tissues or cultured cells were lysed in IP buffer supplemented with protease inhibitors. Lysates were precleared with Protein A/G beads and then incubated with specific antibodies against H3K18la or GATA4 overnight at 4°C with gentle agitation. Protein–antibody complexes were captured using Protein A/G beads, washed extensively, and eluted by boiling in SDS sample buffer. Eluted proteins were resolved by SDS‐PAGE and stained with Coomassie Brilliant Blue or transferred for immunoblotting. For mass spectrometry analysis, Co‐IP samples were subjected to trypsin digestion, and peptides were analyzed using LC–MS/MS to identify interacting proteins.

### 4.10 Luciferase Reporter Assay

To assess transcriptional activity, NRCMs were transfected with a luciferase reporter construct containing the ANP promoter region using Lipofectamine 2000 reagent (Thermo Fisher Scientific). Cells were cotransfected with plasmids expressing GATA4 and Renilla luciferase for normalization. After 24 h, cells were lysed, and luciferase activity was measured using a dual‐luciferase reporter assay system (Promega). Firefly luciferase activity was normalized to Renilla luciferase activity to determine relative promoter activity.

### 4.11 Assessment of Glycolytic Capacity via Seahorse Extracellular Acidification Rate Analysis

To assess glycolytic metabolism, extracellular acidification rate (ECAR) was measured in live cardiomyocytes using the Seahorse XFe96 Analyzer (Agilent Technologies). Briefly, primary cardiomyocytes were seeded at a density of 2 × 10⁴ cells/well in XF96 cell culture microplates and treated with PE (50 µM) for 24 h to induce hypertrophic stimulation. Prior to the assay, cells were equilibrated for 1 h in XF assay medium (pH 7.4) supplemented with 2 mM glutamine and 10 mM glucose under non‐CO_2_ conditions. Sequential injections of glycolytic pathway modulators were performed: (1) 10 mM glucose to measure basal glycolysis, (2) 1 µM oligomycin (ATP synthase inhibitor) to induce maximal glycolytic capacity, and (3) 50 mM 2‐deoxyglucose (hexokinase inhibitor) to confirm glycolysis‐dependent acidification. ECAR values (mpH/min) were normalized to total protein content quantified via BCA assay. Glycolytic parameters (basal glycolysis, glycolytic capacity, and glycolytic reserve) were calculated as previously described [55].

### 4.12 Statistical Analysis

Data are presented as mean ± standard error of the mean from at least three independent experiments. Statistical significance was determined using Student's *t*‐test for comparisons between two groups or one‐way analysis of variance followed by Tukey's post hoc test for multiple comparisons. A *p* value <0.05 was considered statistically significant.

## Author Contributions

L. Hu, H.D. Guo, and D.Y. Fu designed the experiments and drafted the manuscript. M.Z. Wang, Z.X. Liu, and Y.B. Ma performed the experiments. Y.N. Wang, D. Li, and Y. Lin helped with the experiments. All authors reviewed the manuscript and approved the submission.

## Conflicts of Interest

The authors declare no conflicts of interest.

## Ethics Statement

This study was approved by the Institutional Review Board of Yueyang Hospital of Integrated Traditional Chinese and Western Medicine, Shanghai University of Traditional Chinese Medicine (2022‐051). Written informed consent was obtained from all participants. All experimental protocols involving animals in this study were approved by the Laboratory Animal Research Committee of Shanghai University of Traditional Chinese Medicine (PZSHUTCM2304240008).

## Supporting information




**Figure S1**: M‐mode echocardiograms of hearts from TAC or a sham operation. LV end‐diastolic volume (LVEDV), LV end‐systolic volume (LVESV), LV internal dimension‐diastole (LVIDd), LV internal dimension‐systole (LVIDs), stroke volume (SV), and cardiac output (CO).
**Figure S2**: M‐mode echocardiograms of hearts from TAC and oxamate treatment. LVEDV, LVESV, LVIDd, LVIDs, SV, and CO.
**Figure S3**: M‐mode echocardiograms of hearts from TAC, oxamate, and AAV‐GATA4 treatment. LVEDV, LVESV, LVIDd, LVIDs, SV, and CO.
**Figure S4**: AKAP2 overexpression did not alert the inhibition of lactylation attenuated the TAC impaired cardiac contractile functions and induced hypertrophy mice. (A) Representative examples of hearts from TAC or a sham operation. (B) The ratio of heart weight to body weight (HW/BW). (C) The ratio of left ventricle weight to tibia length (LV/TL).
**Table S1**: Baseline characteristics of the patients and health donors (*n* = 20).
**Table S2**: Primers for real‐time reverse transcriptase polymerase chain reaction.

## Data Availability

The data that support the findings of this study are available from the corresponding author upon reasonable request.
