# Supplementary material for: Histone H3 Lysine 18 Lactylation Promotes Cardiac Hypertrophy Through Activating GATA Binding Protein 4 Signaling
Source: MedComm (2020). 2025 Oct 22;6(11):e70421. doi: 10.1002/mco2.70421 (PMC12547076; doi:10.1002/mco2.70421)
Supplement: Supplementary file 1 — Figure S1: M‐mode echocardiograms of hearts from TAC or a sham operation. LV end‐diastolic volume (LVEDV), LV end‐systolic volume (LVESV), LV internal dimension‐diastole (LVIDd), LV internal dimension‐systole (LVIDs), stroke volume (SV), and cardiac output (CO). Figure S2: M‐mode echocardiograms of hearts from TAC and oxamate treatment. LVEDV, LVESV, LVIDd, LVIDs, SV, and CO. Figure S3: M‐mode echocardiograms of hearts from TAC, oxamate, and AAV‐GATA4 treatment. LVEDV, LVESV, LVIDd, LVIDs, SV, and CO. Figure S4: AKAP2 overexpression did not alert the inhibition of lactylation attenuated the TAC impaired cardiac contractile functions and induced hypertrophy mice. (A) Representative examples of hearts from TAC or a sham operation. (B) The ratio of heart weight to body weight (HW/BW). (C) The ratio of left ventricle weight to tibia length (LV/TL). Table S1: Baseline characteristics of the patients and health donors (n = 20). Table S2: Primers for real‐time reverse transcriptase polymerase chain reaction. [file MCO2-6-e70421-s001.docx]

**Histone H3 lysine 18 lactylation promotes cardiac hypertrophy through activating GATA binding protein 4 signaling**

**Running title：**H3K18la/GATA4 promotes cardiac hypertrophy

Mingzhu Wang^1#^ Zixian Liu^1#^ Yongbo Ma^1#^ Da Li^1^ Yuan Lin^2^  Yani Wang^1^ Deyu Fu^1^* Haidong Guo^2^* Liang Hu^2^*

^1^Yueyang Hospital of Integrated Traditional Chinese and Western Medicine, Shanghai University of Traditional Chinese Medicine, Shanghai, China, 200437

^2^Academy of Integrative Medicine, Shanghai University of Traditional Chinese Medicine, Shanghai, China, 201203

^#^These authors contributed equally to this work.

*Correspondence to Deyu Fu, Haidong Guo and Liang Hu

**
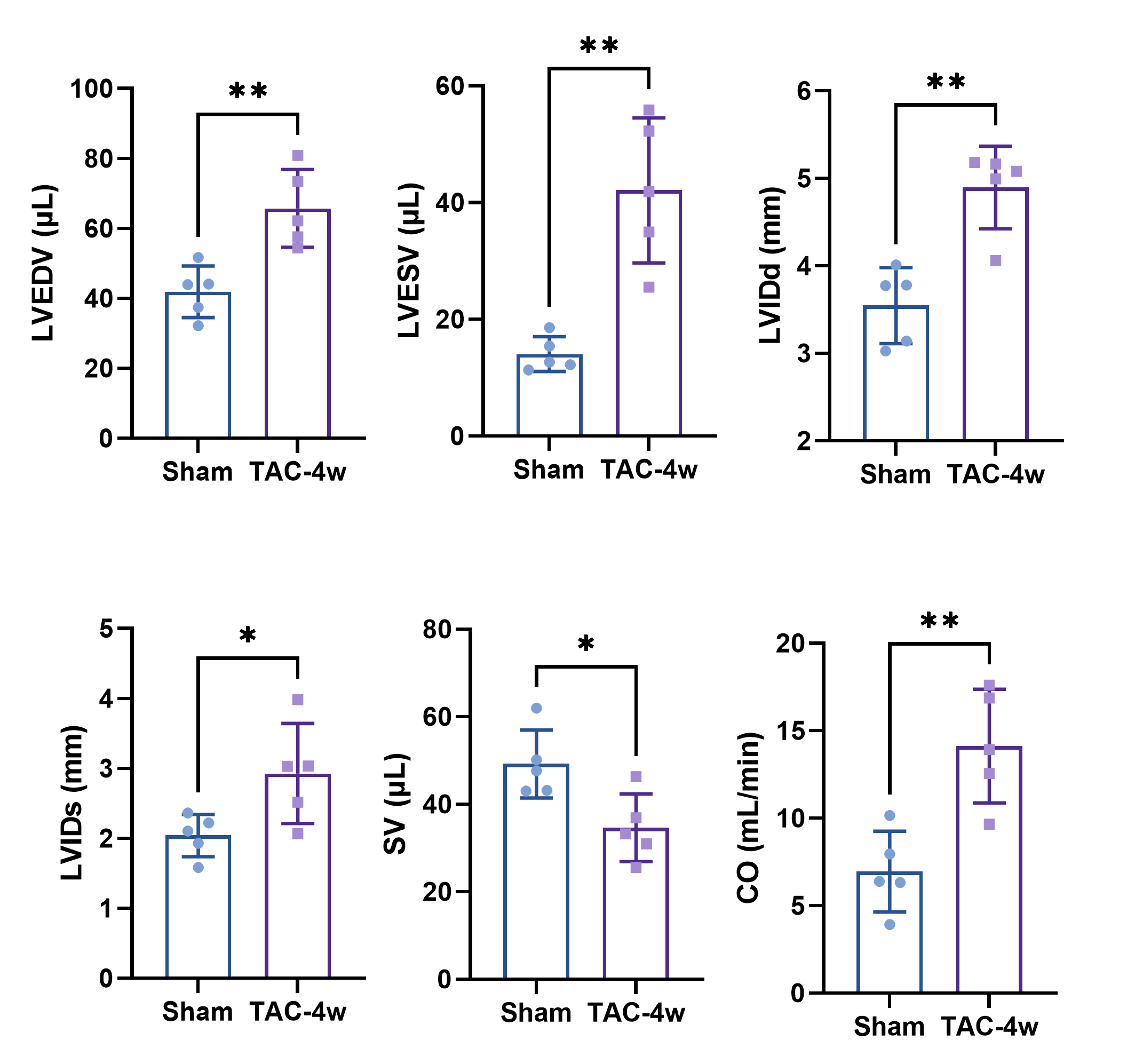
**

**Figure S1.** M-mode echocardiograms of hearts from TAC or a sham operation. LV end-diastolic volume (LVEDV), LV end-systolic volume (LVESV), LV internal dimension-diastole (LVIDd), LV internal dimension-systole (LVIDs), stroke volume (SV), and cardiac output (CO).

**
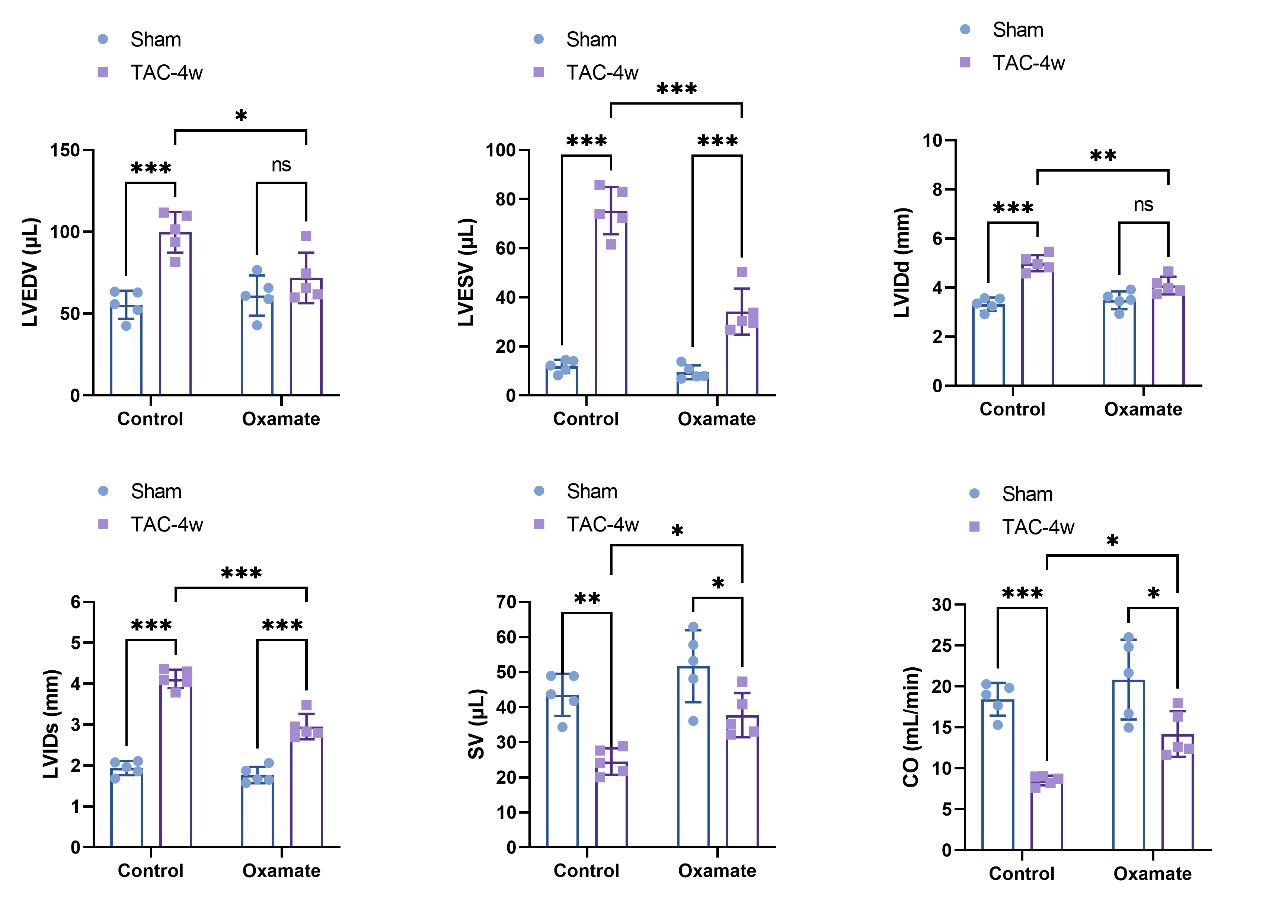
**

**Figure S2.** M-mode echocardiograms of hearts from TAC and Oxamate treatment . LVEDV, LVESV, LVIDd, LVIDs, SV, and CO.

**
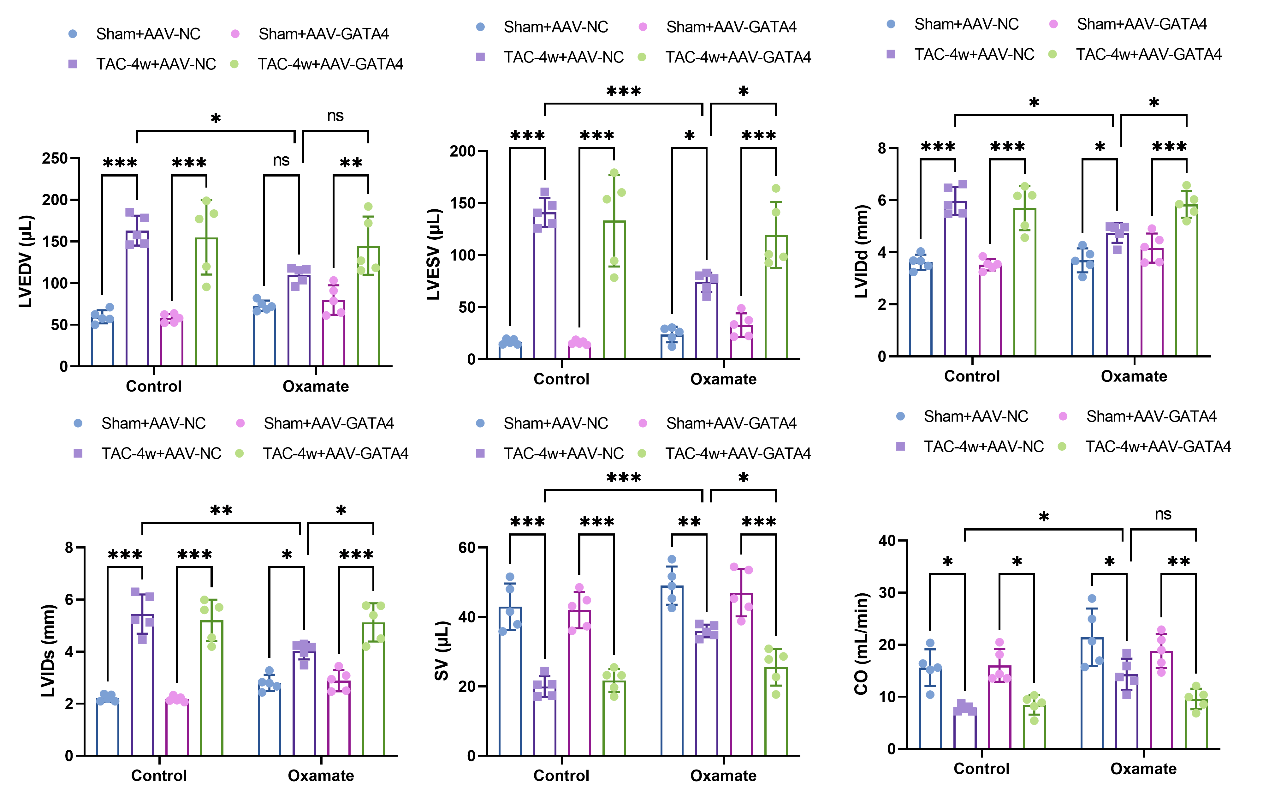
**

**Figure S3.** M-mode echocardiograms of hearts from TAC, Oxamate, and AAV-GATA4 treatment . LVEDV, LVESV, LVIDd, LVIDs, SV, and CO.

**
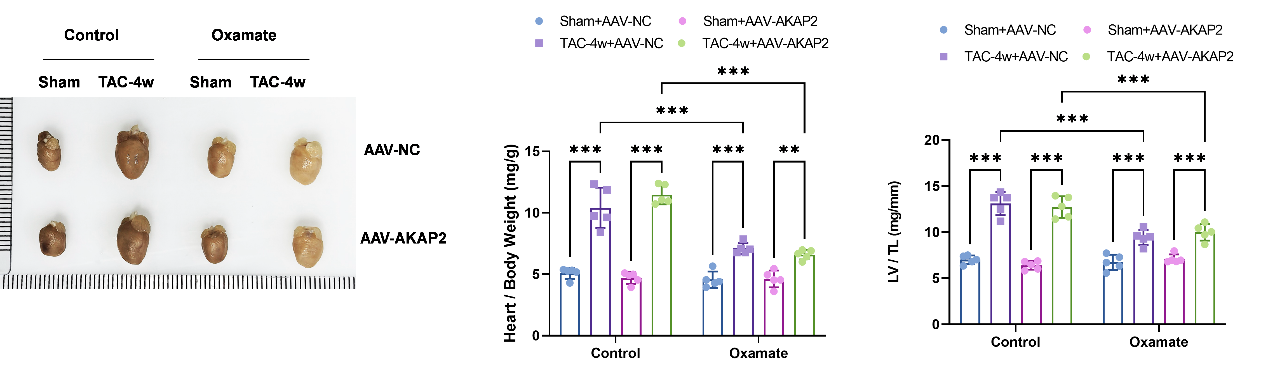
**

**Figure S4.** AKAP2 over-expression did not alert the inhibition of lactylation attenuated the TAC impaired cardiac contractile functions and induced hypertrophy mice. (A) Representative examples of hearts from TAC or a sham operation. (B) The ratio of heart weight to body weight (HW/BW). (C) The ratio of left ventricle weight to tibia length (LV/TL).

**Table S1.** Baseline characteristics of the patients and health donors (n = 20)

| **Characteristics** | **Patients**  **(n=20)** | **Health donors (n=20)** | ***p* value** |
| --- | --- | --- | --- |
| Age, y | 57.9±11.6 | 50.2±13.6 | 0.064 |
| Male sex, n (%) | 12 (60.0) | 11 (55.0) | 0.999 |
| Current smoking, n (%) | 9 (45.0) | 6 (30.0) | 0.515 |
| SBP, mmHg | 147.9±6.01 | 130.4±8.20 | ＜0.001* |
| DBP, mmHg | 90.3±7.93 | 77.35±6.58 | ＜0.001* |

* statistically significant (P<0.05)

**Table S2.** Primers for real-time reverse transcriptase polymerase chain reaction

| **Genes** | **Forwart primer (5’-3’)** | **Reverse primer (5’-3’)** |
| --- | --- | --- |
| Mouse GATA4 | CACCCCAATCTCGATATGTTTGA | GCACAGGTAGTGTCCCGTC |
| Mouse ANP | GTGCGGTGTCCAACACAGAT | TCCAATCCTGTCAATCCTACCC |
| Mouse BNP | GAGGTCACTCCTATCCTCTGG | GCCATTTCCTCCGACTTTTCTC |
| Mouse β-MHC | CCTGCGGAAGTCTGAGAAGG | CTCGGGACACGATCTTGGC |
| Mouse β-actin | GTGACGTTGACATCCGTAAAGA | GCCGGACTCATCGTACTCC |
| Rat GATA4 | ACGGAAGCCCAAGAATCTGA | ATAGTGAGATGACAGCCCGG |
| Rat ANP | GGAAGTCAACCCGTCTCAGA | TGGGCTCCAATCCTGTCAAT |
| Rat BNP | CCGGATCCAGGAGAGACTTC | AGAGCTGGGGAAAGAAGAGC |
| Rat β-MHC | CCAGTCCCGAGGTGTACTTT | TCCTCCTTCATGTTGGCCAT |
| Rat β-actin | TCTTCCAGCCTTCCTTCCTG | CACACAGAGTACTTGCGCTC |
